# Correction

**DOI:** 10.1080/12298093.2023.2239652

**Published:** 2023-08-03

**Article title:** Diversity of the Bambusicolous Fungus *Apiospora* in Korea: Discovery of New *Apiospora* Species

**Authors:** Kwon, S. L., Cho, M., Lee, Y. M., Lee, H., Kim, C., Kim, G.-H., & Kim, J.-J.

**Journal:** *Mycobiology*

**Bibliometrics:** Volume 50, Number 5, pages 302–316

**DOI:** https://doi.org/10.1080/12298093.2022.2133808

Following the publication of the original article (Kwon et al. 2022), we were notified that two novel *Apiospora* species (*Ap. lageniformis* S.L. Kwon & J.J. Kim and *Ap. pseudohyphopodii* S.L. Kwon & J.J. Kim) were invalidly published. For both names two numbers referring to two different culture collections were given as holotype (Art. 40.7, https://www.iapt-taxon.org/nomen/pages/main/art_40.html). Moreover, a statement that the types are preserved in a metabolically inactive state was missing (Art. 40.8) which rendered the names invalid.

They are validated herein.

***Apiospora lageniformis*** S.L. Kwon & J.J. Kim, sp. nov. MB 849424

For a detailed description see Kwon et al., Mycobiology 50(5): 304 (2022).

Holotype: NIBRFGC000509393, preserved in a metabolically inactive state.

***Apiospora pseudohyphopodii*** S.L. Kwon & J.J. Kim, sp. nov. MB 849425

For a detailed description see Kwon et al., Mycobiology 50(5): 304 (2022).

Holotype: NIBRFGC000509202, preserved in a metabolically inactive state.

